# Reproductive Intentions and Outcomes among Women on Antiretroviral Therapy in Rural Uganda: A Prospective Cohort Study

**DOI:** 10.1371/journal.pone.0004149

**Published:** 2009-01-08

**Authors:** Jaco Homsy, Rebecca Bunnell, David Moore, Rachel King, Samuel Malamba, Rose Nakityo, David Glidden, Jordan Tappero, Jonathan Mermin

**Affiliations:** 1 Centers for Disease Control and Prevention (CDC), Global AIDS Program, National Center for HIV, Viral Hepatitis, STD, and TB Prevention, Entebbe, Uganda; 2 Global Health Sciences, Prevention and Public Health Group, University of California San Francisco, San Francisco, California, United States of America; 3 British Columbia Centre for Excellence in HIV/AIDS, University of British Columbia, Vancouver, Canada; Tulane University, United States of America

## Abstract

**Background:**

Antiretroviral therapy (ART) may influence the biological, social and behavioral determinants of pregnancy in HIV-infected women. However, there are limited longitudinal data on the reproductive intentions and outcomes among women on ART in Africa.

**Methodology /Principal Findings:**

Using a prospective cohort design, we analyzed trends in desire for children and predictors of pregnancy among a cohort of 733 HIV-infected women in rural Uganda who initiated ART between May 2003 and May 2004 and were followed up in their homes until June 2006. Women answered in-depth social and behavioral questionnaires administered every quarter in year 1 after initiating ART, and every 6 to 12 months thereafter. Use of family planning methods was assessed at 18 and 24 months after starting ART. We tested for non-constant pregnancy incidence by using a shape parameter test from the Weibull distribution. We modeled repeated measurements of all variables related to the women's desire for children over time using a generalized estimating equation (GEE) extension to the logistic regression model. Risk factors for pregnancy were examined using Cox proportional hazards model. 711 women eligible for the study were followed-up for a median time of 2.4 years after starting ART. During this time, less than 7% of women reported wanting more children at any time point yet 120 (16.9%) women experienced 140 pregnancies and pregnancy incidence increased from 3.46 per 100 women-years (WY) in the first quarter to 9.5 per 100 WY at 24 months (p<0.0001). This was paralleled by an increase in the proportion of women reporting sexual activity in the past 3 months, from 24.4% at baseline to 32.5% over 24 months of follow-up (p = 0.001). Only 14% of women used permanent or semi-permanent family planning methods by their second year on ART. In the multivariate model, younger age (HR = 2.71 per 10-year decrease, 95% CI: 2.95–3.78), having a BMI>18.5 (HR = 1.09, CI: 1.01–1.18) and not having used condoms consistently in the last 3 months (HR = 1.79, CI: 1.02–3.13) were independently associated with pregnancy.

**Conclusion/Significance:**

Women on ART and their partners should be consistently counseled on the effects of ART in restoring fertility, and offered regularly free and comprehensive family planning services as part of their standard package of care.

## Introduction

Antiretroviral therapy (ART) restores health and fertility in people living with HIV and drastically reduces mother-to-child transmission (MTCT) of HIV [1], [2], [3]. As major efforts are under way to expand access to this life-saving treatment in sub-Saharan Africa [4], [5], thousands of men and women on ART are resuming a socially productive and sexually active lives involving protected and unprotected sex with or without a desire for children [2], [6], [7], [8].

Numerous behavioral and contextual factors interact in a complex way to determine intended and unintended reproductive outcomes among women living with HIV. Age, marital, educational, and socio-economic status, cultural and religious beliefs, sexual behavior as well as family size and losses, and access to family planning services are documented predictors of pregnancy [9], [10], [11], [12], [13]. In sub-Saharan Africa where HIV prevalence is highest, these factors may be considerably influenced by the traditional roles of women, the socio-cultural importance of motherhood, and a woman's partner's desire for children independent of her own [14], [15], [16], [17]. The added risk of MTCT of HIV through breastfeeding [18] and a reduced capacity for timely diagnosis and treatment of infants infected with HIV [19] compounds the complexity of the choices that couples or women living with HIV must make in relation to childbearing.

ART adds a new dimension to this situation, challenging people living with HIV/AIDS (PLHA), their partners and their care providers to address the impact of ART on their fertility, sexual behavior and reproductive decisions. Recent cross-sectional surveys and qualitative studies have shown that women on ART in sub-Saharan resume their love and sexual life once their health is restored and want to have children [7], [8], [20]. This study examined the reproductive intentions and outcomes over 2 years of follow-up among 733 HIV-infected women on ART enrolled in a prospective cohort study in rural Uganda.

## Methods

### Objectives

The specific objectives of this study were 1) to compare the baseline characteristics of HIV-infected women who did or did not became pregnant following their initiation of ART, 2) to analyze trends in pregnancy incidence, desire for children and sexual activity over time among these women following ART initiation, 3) to describe the rate of unmet need for family planning and the rate of contraceptive use among these women after 2 years of follow-up, and 4) to assess independent risk factors for pregnancy over 2 years of follow-up post ART initiation.

### Participants

**Figure 1:**
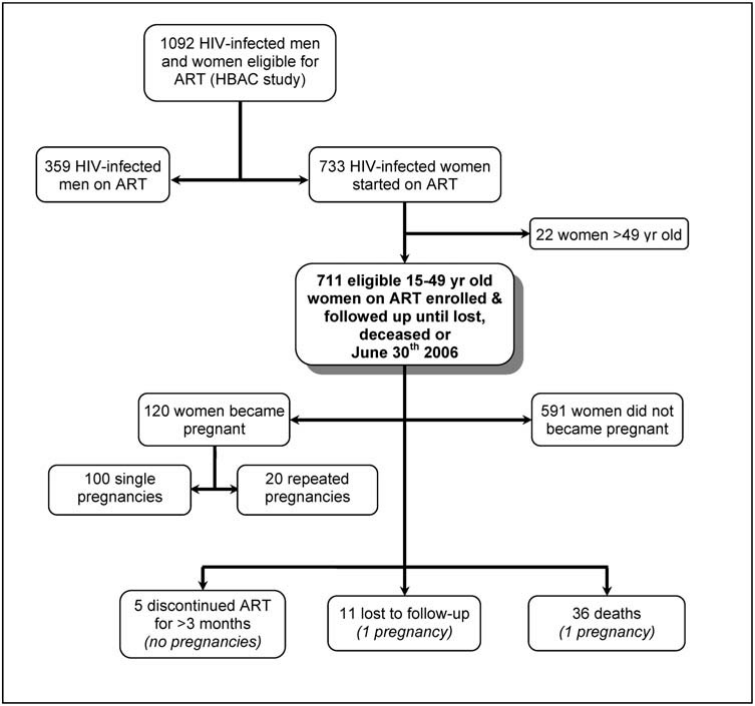
HBAC pregnancy sub-study enrolment and follow-up flow chart.

Women included in this analysis were part of the Home-Based AIDS Care (HBAC) study, a randomized trial of three different ART monitoring strategies involving 1,092 HIV-infected men and women in Tororo and Busia districts in rural Eastern Uganda [15] (Figure 1). HBAC study participants were recruited among clients of the Tororo branch of The AIDS Support Organization (TASO), an indigenous non-governmental organization providing medical and psycho-social care and support for PLHA. Starting in March 2003, TASO clients ≥18 years-old were screened for ART eligibility and were offered first-line ART if they had a CD4 cell count <250 cells/µL or were in WHO disease stage 3 or 4. All households participating in the study received insecticide-treated bed nets to reduce malaria incidence and a water vessel with a supply of chlorine for diarrheal disease prevention. All household members were offered home-based HIV counseling and testing; 99% of them accepted and those diagnosed with HIV were offered couple counseling, were provided with free care and treatment, and were offered enrollment in HBAC [21]. Among HBAC participants, 97% of sexually active women and 98% of sexually active men had disclosed their HIV sero-status to a median of 15 people at baseline, including their sexual partner (Nakayiwa S. et al., unpublished data), reflecting TASO's long-standing ‘positive living’ philosophy which includes empowering PLHA to disclose their HIV sero-status [22].

### Enrolment and follow-up procedures – HBAC study

As part of the enrollment into HBAC, participants underwent physical examinations and answered in-depth questionnaires to evaluate their social, behavioral, and economic baseline characteristics as well as clinical history. Study participants were counseled at enrolment about the potential effects of ART on improving health status, restoring fertility and sexual activity, and were offered referral to the district hospital family planning (FP) clinic that was adjacent to the study clinic if interested in using any FP method other than condoms. The hospital FP clinic provided standard FP counseling and free FP services including any number of clinic visits and contraceptives supplied by the Ministry of Health including combined oral and progesterone-only pills, hormonal injectables and implants, intra-uterine devices, tubal ligation, and condoms. Condoms were also available for free from all HBAC clinicians, counselors and home visitors, both at the study clinic and during home visits.

After enrollment, HBAC participants were visited in their homes on a weekly basis by trained lay workers who delivered drugs, conducted pill counts and monitored participants' clinical status using standardized checklists. No routine clinic visits were scheduled but participants could come to the clinic or hospital when experiencing acute symptoms or showing signs of potential drug toxicity or disease progression. There were no clinic visits fees or any other charge associated with the care provided by HBAC. Home visitors weighed participants and asked women for the date of their last menstrual period on a monthly basis. Women were tested for pregnancy if they had missed their periods for more than 4 weeks. Home visitors also drew blood from participants on a quarterly basis for measurement of CD4 count and viral load. HBAC counselors visited participants quarterly in their homes to provide systematic confidential counseling for psycho-social support, treatment adherence and prevention of sexual transmission of HIV including safer sex and family planning counseling and condoms.

Counseling sessions were carried out in the participants' native language and lasted on average 30 minutes. Counselors also administered in-depth questionnaires related to social and behavioral factors including sexual practices at baseline and quarterly in the first year of follow-up, bi-annually in year 2, and annually in year 3, except for a module on HIV transmission knowledge and desire for children which was administered annually as of year 2. These interviews lasted on average 1 hour and were also carried out in the participants' own home and own language. Women's partners were not interviewed unless they were an HBAC index client. In this study, the partners' desire for children was reported through the women.

The team of home visitors consisted in 30 lay workers trained by HBAC investigators in basic counseling, clinical and adherence monitoring, and referral. The team of counselors consisted in 12 certified and experienced professional counselors trained by HBAC investigators in research methods, behavioral and counseling theory, and family planning in relation to HIV/AIDS. Each home visitor and counselor was assigned to a given household for the entire duration of the study in order to increase the trust of the participants and enhance the validity of the information collected throughout the study. The frequency of home visits and the counseling messages remained the same over the course of the study. The original HBAC study duration was 36 months (median cohort follow-up) but this cohort had taken part in two preceding home-based studies [23], [24], thus most counselors and home visitors had been with their assigned household for over a year at the time the HBAC study started.

### Enrolment and follow-up procedures - pregnancy sub-study

All female HBAC participants 18–49 years old who initiated ART between March 2003 and May 2004 were included in the pregnancy sub-study. Pregnancies occurring during follow-up were identified through clinic and home visits and were confirmed by one or more of the following criteria: a positive human chorionic gonadotropin test, a positive obstetrical examination, the presence of fetal heart beat, a history of abortion, or the delivery of a baby.

Pregnant women were educated about giving single-dose nevirapine prophylaxis to their newborn babies for the prevention of MTCT of HIV and were counseled to exclusively breastfeed for 3 to 6 months followed by rapid weaning as per Uganda's national PMTCT guidelines at the time [25]. Pregnancy outcomes were recorded by field and clinical staff. Post-conception counseling did not provide or refer clients for pregnancy termination services as voluntary abortion is illegal in Uganda. However, women who pursued termination on their own were asked about their experiences and were treated for any complications.

### Ethics

All participants provided written consent to participate in the HBAC study in one of six local languages. The HBAC study and the pregnancy sub-study were approved by the Science and Ethics Committee of the Uganda Virus Research Institute, the Uganda National Council of Science and Technology, and the institutional review boards of the Centers for Disease Control and Prevention (CDC), Atlanta, Georgia, USA, and of the University of California, San Francisco, California, USA.

### Statistical methods

Women with a confirmed pregnancy and whose last menstrual period occurred at least one month after the date of ART initiation were considered to have become pregnant while on ART. Women who were pregnant at the time of starting ART were enrolled 1 month after the end of their pregnancy. Assessment of the last normal menstrual period date was based on self-report, assessment of fundus height, or back dating from actual date of delivery of a live baby. Women who discontinued ART for more than 3 continuous months were excluded from the analysis during the time of treatment discontinuation. Women who did not become pregnant were censored at the date of discharge or on June 30^th^ 2006. Women who became pregnant were censored at the estimated date of conception. Conception date was estimated at 15 days after the date of the last reported or estimated normal menstrual period. Based on these criteria, 711 of 733 women enrolled in the HBAC trial were eligible for this study (Figure 1). Three of these women were missing baseline data and thus were excluded from the baseline and multivariate analyses.

We compared the baseline characteristics of women who did or did not became pregnant using the Wilcoxon rank sum test for continuous variables and the Chi-squared test for categorical variables. We analyzed trends in pregnancy incidence and desire for children over time. We tested for non-constant incidence of pregnancy by using a test for the shape parameter from the Weibull distribution [26]. We modeled repeated measurements of all variables related to the women's desire for children over time using a generalized estimating equation extension (GEE) to the logistic regression model [27]. The p-values were derived from a chi-square test of a linear trend arising out of the GEE model. We examined the issue of linearity by performing a test for departure from linear trend which was not significant. We also described family planning practices and the unmet need for family planning services in the second year of follow-up.

Risk factors for pregnancy were examined using the Cox proportional hazards model [28]. We assessed the unadjusted effects of potential risk factors for the first incident pregnancy using the log rank test [29]. Subsequent pregnancies were not included in the bi- and multivariate analyses. Adjusted predictor effects were summarized using hazard ratios (HR) from the multivariable models. Most predictor variables were assessed at multiple time points during follow-up. We treated these variables as time-dependent covariates [30] unless otherwise specified. At a given time point, the value of the predictor variable was determined by the most recent prior measurement. All p-values are two-sided. All analyses were conducted in Stata version 9.0 (Stata Corporation, College Station, Texas, USA).

## Results

### Enrolment and baseline characteristics (Figure 1 & Table 1)

**Table 1:** Baseline biological, socio-economic, and behavioral characteristics of women on ART (N = 708*).

Socio-economic status		Pregnant after ART		NOT pregnant after ART		p-value
		N = 117*	% or (IQR)	N = 591	% or (IQR)	
Median age (years)		32 (30–36)		38 (32–42)		**<0.0001**
Median follow-up (years)		2.40 (2.29–2.56)		2.46 (2.33–2.63)		0.10
Education	None	28	25%	141	24%	0.58
	Primary	65	57%	313	53%	
	Post-primary	21	18%	134	23%	
Religion	Christian	110	97%	557	95%	0.52
	Moslem	4	3.5%	25	4.3%	
	Other	0	0%	6	1.0%	
Marital Status	Single	8	7%	24	4.1%	**0.002**
	Married / co-habiting	46	40%	148	25%	
	Separated / divorced	13	11%	65	11%	
	Widowed	48	42%	350	60%	
Income	Farming	33	29%	183	31%	0.93
	Trade	41	35%	184	31%	
	Dependent	21	18%	101	17%	
	Wages & others	17	15%	100	18%	
	None	3	3%	19	3%	
**Family situation and desire for children**						
Has biological children		115	99%	568	97%	0.55
Total no. of live children	0	3	2.6%	20	3.5%	0.45
	1–2	36	31%	147	26%	
	3+	76	66%	402	71%	
Total no. of deceased children	0	28	24%	192	34%	0.13
	1–2	60	52%	266	47%	
	3+	27	24%	109	19%	
Experienced death of child	ever	86	74%	341	60%	**0.004**
	in last 12 months	8	9.3%	25	7.4%	0.55
Experienced death of spouse/partner	ever	63	54%	382	66%	**0.014**
	in last 12 months	9	14%	28	7.3%	0.065
Experienced marriage break-up	ever	56	49%	241	42%	0.16
	in last 12 months	9	16%	15	6.3%	**0.025**
Wants more children		5	4.4%	17	2.9%	0.39
Planning to have more children		1	1.0%	9	1.9%	0.47
Partner wants more children **		13	22%	39	19%	0.63
**Clinical status**						
WHO Staging	1–2	72	62%	329	56%	0.24
	3–4	45	38%	262	44%	
Body mass index	<18.5	30	26%	138	24%	0.21
	18.5–24.9	81	70%	391	68%	
	≥25	4	3.5%	47	8.2%	
CD4 count	≤50	21	18%	100	17%	0.95
	>50 to 200	66	56%	333	56%	
	>200	30	26%	158	27%	
	Median (IQR)	141 (71–204)		138 (76–204)		0.99
Pregnant	at baseline	4	3.8%	11	1.9%	0.27
	in last 12 months	15	13%	18	3.1%	**<0.0001**
Trial arm (ART monitoring):	Clinical+CD4+viral load	40	34%	195	33%	0.86
	Clinical+CD4 count	43	37%	205	35%	
	Clinical only	34	29%	191	32%	

Sexual behavior		Pregnant after ART		NOT Pregnant after ART		p-value
		N = 117	% or (IQR)	N = 591	% or (IQR)	
Had sex in past 3 months		48	42%	121	21%	**<0.0001**
No. of sex partners in last 3 months	0	6	11%	54	31%	**0.008**
	1	47	89%	121	67%	
	2+	0	0%	3	2%	
Type of current sexual partner	Spouse	30	63%	82	68%	0.79
	Steady	13	27%	27	22%	
	Casual	5	10%	12	10%	
Partner HIV status ***	HIV-negative	24	21%	62	10%	0.30†
	HIV-positive	22	19%	81	14%	
	unknown	71	61%	448	76%	
Partner on ART ***		16	14%	52	8.8%	0.10
Ever experienced physical abuse by partner		56	49%	259	44%	0.41
Not worried about transmitting HIV to sexual partner		50	43%	253	44%	0.90
Time since started having sex with recent partner (years)	Median (IQR)	4.8 (1.1–13.3)		8.3 (2.5–16.2)		0.11
Sex frequency with current partner [median (IQR)]:	in past week	1.0 (1–2)		2.0 (1–2)		0.23
	in past month	2.0 (1–3)		2.0 (1–2)		0.14
Always used condom in last 3 months		28	60%	67	57%	0.74
Used condom at last sex		31	65%	72	60%	0.58

* Three women did not have baseline data.

** Among 262 women who answered this question.

*** Partners' HIV or ART status near the time of conception was used for women who became pregnant and by mid-follow-up (or approx. ∼12 months) for women who did not become pregnant, which corresponded to the median time to first incident pregnancy among women who became pregnant.

† excluding unknown.

Women enrolment is summarized in Figure 1 and enrolled women's characteristics are described in Table 1. 711 women were enrolled and followed up for a median of 2.44 years (interquartile range (IQR): 2.32–2.62); of these, 11 (1.6%) were lost to follow-up, 5 (0.6%) discontinued ART for >3 months, and 36 (5.1%) died. Among 708 women with baseline data, median age was 37 years (IQR 31–41), 28% (194/702) of all women were married or co-habiting with their partner, 57% (398/702) were widowed, 24% (169/702) had no formal education, and 97% (661/684) had one or more living children (median 4 children (IQR: 1–5). Twenty-four percent (169/694) of women reported being sexually active at baseline, 2% (3/168) of whom reported having more than 1 current sexual partner, and 56% (95/169) reported having always used a condom with their sexual partner in the last 3 months. Forty-five percent (315/697) of the women reported ever having been physically abused by their partner (Table 1).

At baseline, women who became pregnant after starting ART were younger than their non-pregnant counterparts (median age 32 vs. 38 years; p<0.0001), more likely to be married and/or co-habiting with their partner (40% vs 25%; p = 0.002), to have been pregnant in the last 12 months (13% vs 3%; p<0.0001), and to have experienced the death of a child (74% vs 60%; p = 0.004), but less likely to have experienced the death of a spouse or partner (54% vs 66%; p = 0.014). Pregnancy was not associated with the women's allocation to the various ART monitoring arms of the HBAC trial (Table 1).

### Desire for children and incidence of pregnancy (Figure 2)

**Figure 2:**
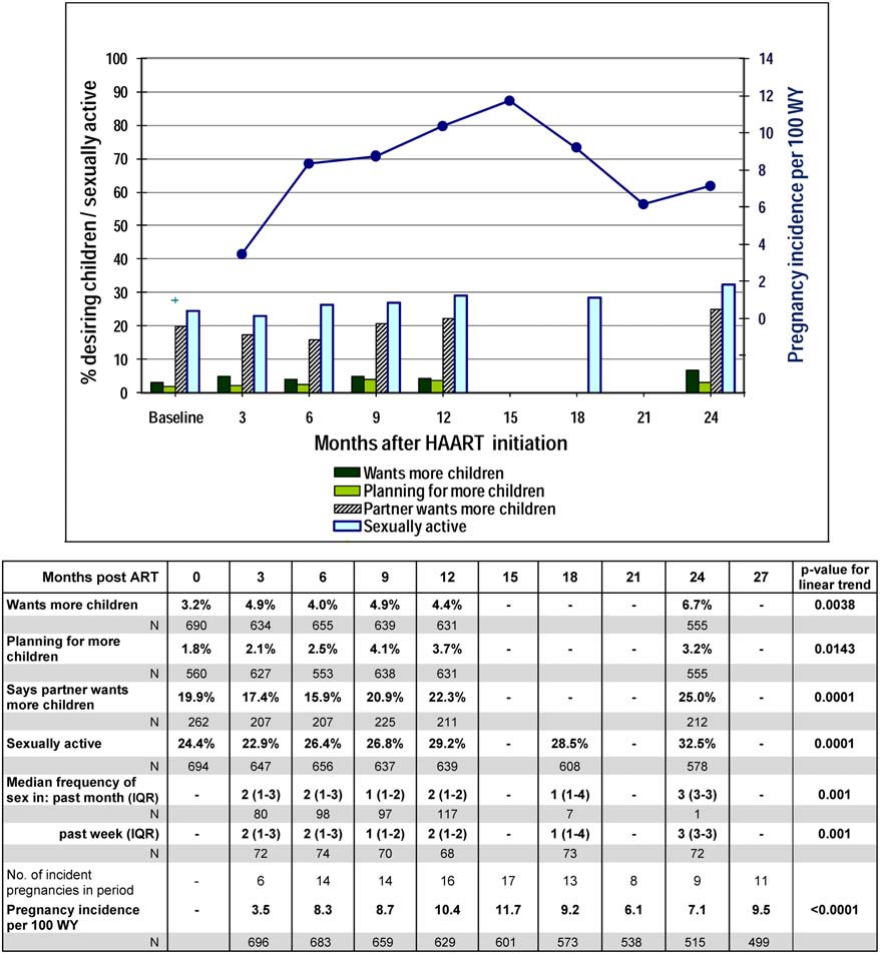
Desire for children and incidence of pregnancy among HBAC women on ART.

Desire for children increased significantly over follow-up for both the women (p<0.004) and their partners (p = 0.0001) but remained under 7% for the women and under 26% for their partners throughout follow-up. The proportion of women reporting sexual activity within 3 months of the interview also increased significantly over time (p = 0.0001), which was reflected by an increased frequency of sex in the past month or the past week (both p-values = 0.001).

A total of 120 (17%) women experienced 140 pregnancies after initiation of ART. Median time to first incident pregnancy was 12.4 months (IQR 7.9–18.0) after starting ART. Four of the140 incident pregnancies produced a set of twins and 38 (26%) did not result in a live birth; of these, 4 were spontaneous miscarriages, 8 were stillbirths including one of the twins, and 26 were induced abortions. Overall pregnancy incidence rate was 8.20 per 100 women-years (WY), increasing from 3.5 per 100 WY in the first quarter post-ART to 11.7 per 100 WY in the fifth quarter of follow-up, and ranging between 6.1 and 9.5 per 100 WY thereafter (p<0.0001) (Figure 2).

### Family planning (Table 2)

**Table 2:** Contraceptive use and unmet need for family planning among sexually active women on ART.

Time after start of ART	18 months		24 months	
**Contraceptive use** (N = sexually active women a)	173 b	%	188 b	%
Not using any contraceptive method c	42	24.3%	59	31.4%
Condom	112	64.7%	119	63.3%
Semi-permanent or permanent method d	24	13.9%	25	13.3%
Dual contraception e	6	3.5%	8	4.3%
Periodic abstinence	7	4.0%	6	3.2%
**Unmet need for FP** (N = sexually active women a not desiring children)	-		176 b	%
Not using any contraceptive method c	-		59	33.5%
Using condoms only	-		93	52.8%
Not using any semi-permanent or permanent method d	-		152	86.4%
Not using dual contraceptive method	-		168	95.5%

a In the last 3 months.

b As respondents could fit in more than one category, percentages add up to more than 100%.

c Not using any semi-permanent or permanent method nor condoms, periodic abstinence or withdrawal.

d Including hormonal methods (pills, injections, implants) and tubal ligation (none of the respondents reported the use of intra-uterine devices).

e Defined as the combined use of a barrier and a hormonal method.

Among sexually active women, 65% (112/173) reported using condoms 18 months after starting ART and 63% (119/188) at 2 years of follow-up. However, only 14% (24/173) and 13% (25/188) reported using a semi-permanent (hormonal injection or implant) or permanent (tubal ligation) contraceptive method at 18 months, and 3.5% (6/173) and 4.3% (8/188) reported using dual contraception (a hormonal and barrier method in combination) after 24 months on ART. Among 176 sexually active women not desiring children at 2 years of follow-up, 33% (59) did not use any method, 53% (93) used condoms alone, 86% (152) were not using any semi-permanent or permanent method, and nearly 96% (168) did not use dual contraception (Table 2).

### Determinants of pregnancy (Table 3)

**Table 3:** Cox proportional hazard analysis of baseline and time-dependent factors associated with pregnancy after ART initiation (N = 117^a^).

Variable	Category or unit	Unadjusted HR	p-value	Adjusted HR	p-value
* **Age**	**per 10-yr decrease**	3.16 (2.30–4.34)	**<0.001**	2.71 (1.95–3.78)	**<0.001**
**Married / cohabitating** *(reference = all single parents* b *)*		2.72 (1.88–3.95)	**<0.001**	1.59 (0.94–2.69)	0.08
Karnofsky score *(reference = 0–80)*	80–100	1.16 (0.29–4.61)	0.84		
**Body mass index**	**≥18.5**	2.50 (1.02–5.88)	**0.037**	1.09 (1.01–1.18)	**0.024**
Time-updated CD4 count	per 50 cells increase	1.001 (1.000–1.002)	**0.045**	1.025 (0.98–1.07)	0.26
* Total no of live children	1–2	1.61 (0.50–5.25)	0.43		
	3+	1.26 (0.40–4.01)	0.69		
* Total no of dead children	1–2	1.35 (0.86–2.12)	0.20		
	3+	1.26 (0.74–2.16)	0.40		
Wants more children		1.37 (0.55–3.41)	0.50		
Planning to have more children		1.01 (0.14–7.30)	0.99		
Partner wants more children		1.42 (0.72–2.78)	0.31		
Experienced death of a child		1.63 (1.06–2.50)	**0.025**	1.94 (0.64–5.95)	0.24
Experienced death of spouse		0.19 (0.04–0.88)	**0.034**	0.49 (0.13–1.83)	0.29
Experienced physical abuse by partner in last 12 months		1.36 (0.91–2.02)	0.13		
Worried about transmitting HIV to spouse/partner		1.30 (0.77–2.19)	0.33		
Type of sexual partner *(reference: spouse)*	Steady	0.90 (0.51–1.59)	0.71		
	Casual	0.43 (0.13–1.43)	0.17		
Length of sexual relationship with most recent partner	per 1 year incr.	0.97 (0.94–0.99)	**0.023**	0.82 (0.65–1.95)	0.31
Frequency of sex with most recent partner in last month *(reference = ≤1)*	2	0.76 (0.26–2.19)	0.61		
	>2	1.36 (0.54–3.42)	0.51		
Used condom in last 3 months with most recent partner *(reference = always)*	Sometimes or never	1.82 (1.05–3.23)	**0.033**	1.79 (1.02–3.13)	**0.041**
	*missing*	*0.76 (0.71–0.85)*	*0.005* c	*0.61 (0.53–0.70)*	*0.003* c

* Variables preceded by an asterisk are baseline values; all others are time-dependent variables updated to the time nearest the date of conception.

a Three women without baseline values were excluded from this analysis.

b Combining single, divorced, separated & widowed.

c Women who reported no sexual activity were not asked about condom use, resulting in a missing value. This significance thus merely reflects the association between sexual activity and pregnancy. This ‘missing’ category was created to account for the fact that 28 (24%) of 117 women with a first incident pregnancy had a routine follow-up interview that did not cover the period of conception (i.e. fell outside the 30 to 90 days period following their last menstrual period).

Factors associated with an increased risk of pregnancy included young age (HR = 3.16 per 10 year decrease, CI: 2.30–4.34, p<0.001), being married or co-habiting with a partner (HR = 2.72, CI: 1.88–3.95, p<0.001), having a body mass index (BMI)≥18.5 near the time of conception (HR = 2.5, CI: 1.02–5.88, p = 0.037), having a higher CD4 cell count near the time of conception (HR = 1.001 per 50 cells increase, CI: 1.000–1.002, p = 0.045), having experienced the loss of a child during follow-up (HR = 1.63, CI: 1.06–2.50, p = 0.025), and having used condoms inconsistently with one's current sexual partner in the last 3 months (HR = 1.82, CI: 1.05–3.23, p = 0.033). Factors associated with a decreased risk of pregnancy included having experienced the death of a spouse during follow-up (HR = 0.19, CI: 0.04–0.88, p = 0.034), and being in a longer relationship with one's current sexual partner (HR = 0.97 per year of relationship, CI: 0.94–0.99, p = 0.023), (Table 3).

In the adjusted model (Table 3), being younger (HR = 2.71 per 10-year decrease in age, CI: 1.95–3.78, p<0.001), having a BMI≥18.5 (HR = 1.09, CI: 1.01–1.18, p = 0.024) and having inconsistently used a condom with the most recent sexual partner in the last 3 months (HR = 1.79, CI: 1.02–3.13, p = 0.041) remained independently associated with pregnancy. Being married or cohabiting with a partner (HR = 1.59, CI: 0.94–2.69, p = 0.08) had a borderline association with pregnancy. However, time-updated CD4 counts (HR = 1.025; CI: 0.98–1.07, P = 0.26), having experienced the death of a child (HR = 1.94; CI: 0.64–5.95, p = 0.24) or a spouse (HR = 0.49, CI: 0.13–1.83, p = 0.29) or the length of the sexual relationship with the most recent partner (HR = 0.82; CI: 0.65–1.95, p = 0.31) were no longer independently associated with pregnancy.

## Discussion

In this cohort of women receiving ART in rural Uganda, sexual activity and incidence of pregnancy significantly increased over follow-up, yet more than 93% of the women repeatedly expressed not wanting or not planning to have more children and said the same was true for more than 75% of their partners. In addition, over 86% of sexually active women not desiring children were not using any modern contraception method other than condoms after 2 years on ART and over 95% of them did not use dual contraception despite having been counseled on family planning at the time of ART initiation and having been offered referral to the local family planning clinic. The rates of desire for children that the women reported for themselves and their partners increased significantly but remained low throughout follow-up and neither increase was significantly associated with pregnancy. In multivariate analyses, only young age, having a BMI≥18.5 and not using condoms consistently in the last 3 months were independently associated with pregnancy.

To our knowledge, these results represent the first of two longitudinal analyses of fertility intentions and outcomes among women on ART in sub-Saharan Africa, together with data from another Ugandan cohort [31]. Our data support observations from industrialized countries that ART restores fertility in women living with HIV [2], [3]. Moreover they agree with recent reports on determinants of pregnancy among women on ART in Africa [31], [32]. However they contrast with recent cross-sectional studies reporting higher rates of desire for children among women and/or couples on ART in sub-Saharan Africa [7], [20]. This may result from the difference in study design as well as reflect the particular characteristics of the HBAC cohort.

The contrast between the low rate of self-reported desire for children and the low rate of contraceptive use by the women in this cohort raises the question of possible bias due to social desirability or stigma. While social desirability cannot be totally ruled out, this low desire for children needs to be considered in the broader context of this study: 1) The HBAC counselors who administered the socio-behavioral questionnaires strived to minimize this bias through establishing a long-term, trustful and non-judgmental relationship with their clients; 2) Based on previous findings from home-based interventions in Uganda including the Apondi et al. study reporting mostly positive social outcomes of home-based ART among HBAC participants [33], [34], stigma is unlikely to have significantly biased the results of the socio-behavioral questionnaires; 3) Overall pregnancy incidence was 8.2 per 100 WY, peaking at 11.7 per 100 WY a year after ART initiation. By comparison, the 2007 US Census Bureau age-specific fertility rate for Ugandan women aged 35 to 39 years old was 19.5 births per 100 WY [35]. Thus, both the overall and peak pregnancy rates following ART initiation in this study remained well below that of the general population throughout follow-up; 4) Perhaps the most convincing argument against a social desirability bias in relation to our findings on the women's reported desire for children is the rate of induced abortions among these women, which was nearly twice that estimated for Eastern Uganda in 2004 [36]. It is likely than an even greater number of women attempted but failed to induce abortions and never disclosed it, as elective abortions are illegal in Uganda. Moreover, considering the risks and costs these women faced in attempting abortion, an unknown proportion may have wanted but never tried to abort. In-depth interviews conducted with a subset of the women confirm this possibility [37]. Thus, our data may well underestimate the actual number of women who wanted and/or attempted to abort; 5) The relatively older age of the women in our cohort and the fact that over two-thirds of them already had 3 or more live children at baseline, may also explain the lack of desire for children among these women. Indeed, although the factors we found to be associated with pregnancy in this study mirrored those reported by related studies [38], [39]; no association was found between pregnancy and the number of children the women had.

After 2 years of follow-up, less than 15% of sexually active women used any modern method of contraception in addition to, or apart from condoms, and less than 5% used dual contraception. These rates were equally low or lower among sexually active women who did not desire children. In comparison, the Uganda 2006 Demographic Health Survey (DHS) reported a rate of condom use of 3.2% and a rate of modern contraceptive use of 18% among sexually active women [40] while the Uganda 2004–05 HIV/AIDS Sero-Behavioral Survey showed that only 7% of sexually active women used a condom at last sex [41]. Thus, although condom use was relatively high among HBAC women, there was a 4- to 6-fold discrepancy between the women's own desire for children and the desire they reported on behalf of their partners, as well as a significantly higher risk of pregnancy for women whose male partners did not use condoms consistently. It is therefore likely that the use of contraceptives in general, and of condoms in particular in this cohort, may have been highly dependent on the women's partners' approval and cooperation, which eventually determined the rate of new pregnancies among these women. Both these assumptions are true in the general and HIV-infected female populations [40], [41], [42], [43] and were further emphasized during a 2008 workshop on the impact of HAART on fertility in sub-Saharan Africa [44]. In addition, many women in this cohort believed that they or their partner's were infertile due to HIV [37], which could have further contributed to their low contraceptive use other than condoms. These factors are being further explored through a qualitative study exploring the perceptions of a subset of these women and their partners about their desire for children, their pregnancy or lack thereof after starting ART, and their knowledge, experiences, intentions and expectations about family planning in relation to ART. These results contrast with those of a recent cross-sectional survey among women on ART in another rural area of Uganda that showed increased use of barrier methods among women on ART [45]. Additional longitudinal studies are warranted to further understand the determinants of contraceptive choice and use among women and couples on ART.

Finally, a high proportion of women in this study reported experiencing physical abuse by their partner at baseline. This rate did not differ from the data on domestic violence reported by the 2006 Uganda Demographic and Health Survey [40], and this variable was not statistically associated with pregnancy in our study. However, given the reported association between domestic violence and child morbidity and mortality [46], HIV care programs in Uganda should provide increased support and protection from physical abuse to their women clients.

### Limitations

This study had several limitations: 1) It was not randomized; 2) Spacing between interviews on sexual behavior varied during follow-up; 3) Information about contraceptive use was only collected as of 18 months of follow-up, thus it was not possible to assess contraceptive use as a determinant of pregnancy. However, the low rate of contraceptive use observed after 18 months of follow-up suggests that contraceptive use among these women was low throughout follow-up, as is the case for Ugandan women in general as mentioned above [40]. This possibility is supported by the results of in-depth interviews that were conducted after this study was completed with a subset of both women who did and did not become pregnant after starting ART [37]; 4) We had no baseline or pre-ART data on the fertility rate of the women or men in this cohort, nor was a comparison possible with a matched group of HIV-infected women not on ART. Therefore we could not assess whether the observed increase in pregnancy incidence over follow-up was due to an increase in either fertility or sexual activity among women on ART, or both. However, the older age of this cohort suggests that ART did have at least a partial effect on fertility, since pregnancy incidence increased even though women were older, had a median of 4 children, and thus were expected to have lower fertility rates and desire for children, independent of their sexual activity or HIV status. At any rate, the fact that this increase remained well below the fertility rate of Ugandan women of the same age may indicate a mere return to ‘normal’ levels of fertility rather than a real increase as compared to HIV-uninfected women or HIV-infected women not on ART; 5) Only 27% of all women had a cohabiting partner at the time of their enrolment into the HBAC program. This rate did not change substantially around the time of conception nor did it differ significantly according to the pregnancy outcomes of the women. Moreover, as partners living outside the women's household were not actively traced or tested by the program, the HIV status of the majority of partners was not known. As well, the type and content of discussions around fertility and pregnancy that may have taken place among couples were not assessed in this study. Thus the potential role of the nature of the couples' relationships, of the partners' HIV status and of the women's knowledge of it in predicting pregnancy could not be analyzed in this study. These limitations highlight the need for more in-depth and controlled research on fertility and reproductive intentions and outcomes among people on ART. Furthermore, they underline the importance of incorporating reproductive health measures into ART study designs, as well as ensuring that ART programs provide regular counseling for, and continued access to, reproductive health services including family planning.

The results of this study prompted initiation of an intervention to increase access to family planning services for both female and male HBAC study participants. As of July 2006, HBAC counselors and nurses were retrained in family planning service delivery for PLHA and started to pro-actively counsel all registered clients on family planning on a quarterly basis, to deliver hormonal contraceptives at home, and to actively refer and follow-up women opting for hormonal implants or tubal ligation to the hospital family planning clinic.

In conclusion, our results emphasize the need and importance of making family planning services an integral and continuous part of ART interventions in Africa. While more research is needed to further explore the factors that may place women on ART at increased risk of unwanted pregnancy, women on ART and their partners should be made clearly aware of the effect of ART on fertility. All clients on ART should be given full and free access to family planning services, including dual methods of contraception [47], [48]. Younger women and couples who do not desire children should be targeted in priority and strongly supported to use effective contraception to avoid unwanted pregnancies. Pro-active family planning counseling should be provided not only at the time of screening patients for ART eligibility but also and more importantly, on a regular basis thereafter, once patients resume their normal sexual activity.
